# The Champion of Homœopathy

**Published:** 1895-04

**Authors:** O. T. Huebner

**Affiliations:** Lancaster, Pa.


### THE CHAMPION OF HOMŒOPATHY.

Lancaster, Pa., March 18th, 1895.

Editor of Homœopathic Physician:—Enclosed please find my check for my subscription to The Homœopathic Physician for 1895.

I am glad to know that you keep The Homœopathic Physician fully up to its original standard of purity. Ever since I have been a subscriber it has championed the cause of pure Homœopathy, and in these days of microbes, anti-toxine, and the hosts of new fads and infallible pharmaceutical preparations, it is more than ever incumbent upon the followers of the master to let our light shine. Success to The Homœopathic Physician. Fraternally yours,

O. T. Huebener.

